# Successfully Overcoming Allergy to Anakinra Through Intravenous Desensitization in a Child “Case Report”

**DOI:** 10.1155/crii/6246850

**Published:** 2025-08-20

**Authors:** Tariq Al Farsi

**Affiliations:** Department of Pediatric Allergy and Clinical Immunology, The Royal Hospital, Muscat, Oman

**Keywords:** Anakinra hypersensitivity, autoinflammatory, child, intravenous desensitization, needle phobia

## Abstract

Anakinra, a recombinant interleukin-1 (IL-1) receptor antagonist, is effective in treating autoinflammatory conditions in children; however, it may elicit a hypersensitive reaction, thus requiring desensitization. We report a case wherein a novel intravenous Anakinra desensitization protocol was applied for a child with hypersensitivity and needle phobia. A 10-year-old girl with tumor necrosis factor receptor-associated periodic syndrome (TRAPS) and Anakinra hypersensitivity underwent a 12-step intravenous desensitization protocol at the Royal Hospital, Oman. Intravenous desensitization was successful without adverse events. Subsequent subcutaneous Anakinra administration was well tolerated without further hypersensitivity or inflammatory episodes. This case demonstrates successful intravenous desensitization against Anakinra in a child with TRAPS and needle phobia, thus enabling continued treatment. This approach may benefit others facing similar challenges and warrants further research.

## 1. Introduction

Anakinra is a recombinant interleukin-1 (IL-1) receptor antagonist comprising 153 amino acids and has a molecular weight of 17.3 kD. IL-1 plays a key role in the inflammatory response; thus, Anakinra has been successfully used to treat conditions, such as rheumatoid arthritis, systemic juvenile idiopathic arthritis, and cryopyrin-associated periodic syndrome [1]. Localized adverse reactions to Anakinra are common, as 50% of patients develop injection-site reactions [2]. However, systemic reactions, including hypersensitivity, are rare. Anakinra desensitization is a recognized option for type 1 (immunoglobulin E [IgE])-mediated hypersensitivity, particularly when alternative therapy is not available [3]. We report a case of a girl with hypersensitivity and needle phobia for whom we successfully applied a novel intravenous Anakinra desensitization protocol.

## 2. Patient Information and Clinical Findings

The patient was a 10-year-old girl with confirmed genetic tumor necrosis factor receptor-associated periodic syndrome (TRAPS), a classic autoimmune genetic disorder characterized by an exaggerated and persistent inflammatory response. Traditional therapy using subcutaneous Anakinra, a prototype IL-1 receptor antagonist, was initiated in May 2023 with a daily single dose of 100 mg. This therapy was administered intermittently, with a course duration of 5–7 days, to control the inflammatory episodes associated with TRAPS that were accompanied by fever, lethargy, headache, lower-limb pains, abdominal pain, vomiting, and elevated inflammatory marker levels. Following the course, her Anakinra use continued in a similar intermittent pattern, reserved for periods of relatively moderate disease flares. In June 2024, the girl experienced an inflammatory episode of TRAPS (lower-limb joint pains, daily undocumented fevers, and abdominal pain), and she started Anakinra at home as planned. However, she developed generalized urticarial rash accompanied by facial puffiness and itchiness within 2 min after receiving the Anakinra injection. In 10 min, she experienced acute coughing, dyspnea, and lethargy with no reported loss of consciousness. This allergic reaction resolved spontaneously at home within 1 h with no active intervention; however, the treating team switched her to oral prednisolone (1 mg/kg/day) the next day. Even after increasing the oral prednisolone dose to 2 mg/kg/day, her inflammatory episodes and symptoms persisted.

## 3. Diagnostic Assessment

In July 2024, she was admitted to the Royal Hospital to discuss other management options. As her symptoms and inflammation remained active (C-reactive protein [CRP]: 195 mg/L, erythrocyte sedimentation rate [ESR]: 130 mm/h, and serum amyloid A: 624 mg/L), she received intravenous methylprednisolone 2 mg/kg/day for 3 days. Allergy skin testing was carried out to confirm the type 1 Anakinra hypersensitivity, which revealed a negative prick test (SPT) using subcutaneous Anakinra 100 mg/0.67 mL as a fluid drop. The SPT results were 5 mm for histamine, 0 mm for the negative control, and 0 mm for Anakinra. The intradermal test (IDT) was prepared as 10% of the subcutaneous Anakinra 100 mg/0.67 mL fluid diluted in normal saline. At 1:10 dilution, the IDT was positive after 20 min, and the wheal size was 15 mm. Immediate or late systemic allergic manifestations were not observed after IDT.

## 4. Therapeutic Intervention

As canakinumab, a human monoclonal antibody that targets and neutralizes IL-1 beta, was not readily available, we planned to attempt subcutaneous Anakinra desensitization following a nine-step (30-min interval) protocol with a total final dose of 100 mg. However, because the girl was needle-phobic and due to the associated discomfort, she refused to enroll in such a painful protocol. Therefore, a novel intravenous 12-step desensitization protocol with a total final dose of 100 mg diluted three times in normal saline was created (Table 1). To prepare for desensitization, the child was admitted to the high-dependency ward for close monitoring. She was already on day 3 of the intravenous methylprednisolone therapy. As a premedication, loratadine (10 mg) and montelukast (5 mg) were administered 1 h before Anakinra infusion. According to the Anakinra desensitization protocol, Anakinra infusion was administered with no allergic reaction occurring within 9 h. Twenty-four hours later, the girl received a regular dose of 100 mg subcutaneous Anakinra without premedication, which was well tolerated with no signs of allergy. In addition, the child received a behavioral support that was crucial in addressing her anxiety about the injections.

## 5. Follow Up and Outcome

Currently, she continues to receive home-based daily subcutaneous Anakinra of 100 mg for >12 months with no reported hypersensitivity reaction. Moreover, she has not reported a subsequent inflammatory episode, and a recent inflammatory marker evaluation (CRP: <4 mg/L, ESR: 29 mm, and serum amyloid A: not repeated) indicated that the inflammatory manifestations of TRAPS were under control. The child and her parents expressed satisfaction with the outcome of the intervention.

## 6. Discussion

Hypersensitivity reactions in children treated with Anakinra are rare; however, when occur may complicate the management of autoinflammatory conditions. Anakinra allergy in children has been observed with intermittent use and dose increments in managing such conditions [4, 5]. Nin-Valencia et al. reported successful continuation of Anakinra after pretreatment in a 3-year-old child who experienced anaphylaxis. However, there is insufficient evidence to ensure repeated positive outcomes with such an approach. Pretreatment with corticosteroids and antihistamines alone with no active desensitization or consideration of alternative treatments for IgE-mediated allergy to Anakinra requires a highly individualized medical decision and a thorough benefit–risk analysis [5]. Needle fear and phobia are important emotional and psychosocial observations associated with vaccination, venipuncture, and injections in children. In a meta-analysis, McLenon and Rogers [6] concluded that needle fear was more frequently encountered in children than in adults, particularly in those with chronic diseases. Children ≤5 years and those aged 10 years old reportedly have needle fear rates of 100% and 65%, respectively, and girls have been found to have a 1.4 times higher risk of needle fear than boys. Without providing psychological interventions and adopting needle-free methods, needle fear and phobia may cause hesitancy and refusal of clinical preventive and treatment interventions involving needle use [6]. The nine-step subcutaneous Anakinra desensitization protocol requires diluting 100 mg of Anakinra in water for injection to make solutions at three concentrations. This was the best possible safe and practical approach (30-min intervals with minimum injections). However, considering that our patient had a needle phobia, which caused her to refuse the existing multiple daily subcutaneous injection regimen, we created a 12-step intravenous Anakinra desensitization protocol that required diluting 100 mg of Anakinra in normal saline to provide solutions at three concentrations. Each bag was light-protected and used within 8 h of preparation. After successful desensitization and providing tailored behavioral support, the child was able to accept a single subcutaneous injection daily with ease. Although this situation has not yet arisen, discontinuing the daily subcutaneous Anakinra will probably require repeated intravenous desensitization once the Anakinra is reused for a flare. To the best of our knowledge, intravenous desensitization to Anakinra in children has not been reported previously. However, subcutaneous desensitization is more widely used in adults and children for both Anakinra and canakinumab hypersensitivity. The use of intravenous Anakinra in children was prevalent during the COVID-19 pandemic as a treatment option for severe and refractory inflammatory response associated with multisystem inflammatory syndrome in children [7]. Yang et al. [8] conducted a retrospective review of 14 critically ill children treated with intravenous Anakinra for cytokine storm syndromes, with no reported adverse events. Phadke et al. [9] described the successful prolonged use of intravenous Anakinra to control macrophage activation syndrome (MAS) in 19 children. The infusion was generally well tolerated, with mild reversible transaminitis observed in one patient. There were no reported cases of anaphylaxis. The maximum dose administered was ≤15 mg/kg/day. However, 100 mg of intravenous Anakinra every 6 h (equivalent to 20 mg/kg/day) was administered to a patient who was already on 100 mg of subcutaneous Anakinra twice daily at home to control his acute MAS [9]. In addition to hyperinflammation severity, the intravenous route is preferred over the subcutaneous route for thrombocytopenia, coagulopathy, or subcutaneous edema in critically ill children. Reporting the outcome of our patient along with the aforementioned evidence of the efficacy and safety of intravenous Anakinra in children opens avenues for further use of this approach in Anakinra hypersensitivity.

Intravenous desensitization to Anakinra is effective for IgE-mediated hypersensitivity, particularly for those with contraindications to the subcutaneous route and for children with needle phobia. This approach may be valuable for other children with similar hypersensitivity reactions and needle phobia in whom subcutaneous desensitization is not feasible. Further investigation is required to assess the efficacy and safety of intravenous Anakinra desensitization in larger pediatric populations.

## Figures and Tables

**Table 1:** A 12-step intravenous desensitization protocol to Anakinra hypersensitivity.

Steps	Dilution	Concentration (mg/mL)	Rate (mL/h)	Time (min)	Volume (mL)	Injected dose (mg)	Cumulative dose (mg)
1	1:100	0.01	1	15	0.25	0.0025	0.0025
2			3	15	0.75	0.0075	0.0100
3			6	15	1.50	0.0150	0.0250
4			9	15	2.25	0.0225	0.0475

5	1:10	0.1	3	15	0.75	0.0750	0.1225
6			6	15	1.50	0.1500	0.2725
7			9	15	2.25	0.2250	0.4975
8			12	15	3.00	0.3000	0.7975

9	1:1	1	6	15	1.50	1.5000	2.2975
10			9	15	2.25	2.2500	4.5475
11			12	15	3.00	3.0000	7.5475
12			15	370	92.5	92.453	~100

*Note:* The final dose of ~100 mg of Anakinra. Normal saline was used for dilution. Dilution 1:100 was made by adding 1 mg into 100 mL, dilution 1:10 was made by adding 10 mg into 100 mL, and dilution 1:1 was made by adding 100 mg into 100 mL. The total duration was 535 min.

## Data Availability

The data that support the findings of this study are available from the corresponding author upon reasonable request.

## References

[1] Pty Ltd I (2015). Attachment 1: Product Information for AusPAR Kineret Anakinra A Menarini Australia Pty Ltd KINERET ® (Anakinra) NAME OF THE MEDICINE.

[2] Kaiser C., Knight A., Nordström D. (2012). Injection-Site Reactions Upon Kineret (Anakinra) Administration: Experiences and Explanations. *Rheumatology International*.

[3] Soyyiğit Ş., Kendirlinan R., Aydın Ö., Çelik G. E. (2014). Successful Desensitization With Anakinra in a Case With Immediate Hypersensitivity Reaction. *Annals of Allergy, Asthma and Immunology*.

[4] Aguiar C. L., Pan N., Adams A., Barinstein L., Lehman T. J. (2015). Anaphylaxis to Anakinra in a Pediatric Patient With Systemic Juvenile Idiopathic Arthritis Successfully Treated With Canakinumab: A Case-Based Review. *Clinical Rheumatology*.

[5] Nin-Valencia A., Murias S., Gómez-Traseira C. (2022). Anakinra Hypersensitivity Reaction in a Paediatric Patient With Autoinflammatory Syndrome. *Rheumatology*.

[6] McLenon J., Rogers M. A. M. (2019). The Fear of Needles: A Systematic Review and Meta-Analysis. *Journal of Advanced Nursing*.

[7] Della Paolera S., Valencic E., Piscianz E. (2021). Case Report: Use of Anakinra in Multisystem Inflammatory Syndrome During COVID-19 Pandemic. *Frontiers in Pediatrics*.

[8] Yang L., Lowry S., Heath T. (2023). Use of Intravenous Anakinra for Management of Pediatric Cytokine Storm Syndromes at an Academic Medical Center. *Hospital Pharmacy*.

[9] Phadke O., Rouster-Stevens K., Giannopoulos H., Chandrakasan S., Prahalad S. (2021). Intravenous Administration of Anakinra in Children With Macrophage Activation Syndrome. *Pediatric Rheumatology*.

